# Tinker-HP: a massively parallel molecular dynamics package for multiscale simulations of large complex systems with advanced point dipole polarizable force fields†
†Electronic supplementary information (ESI) available. See DOI: 10.1039/c7sc04531j


**DOI:** 10.1039/c7sc04531j

**Published:** 2017-11-27

**Authors:** Louis Lagardère, Luc-Henri Jolly, Filippo Lipparini, Félix Aviat, Benjamin Stamm, Zhifeng F. Jing, Matthew Harger, Hedieh Torabifard, G. Andrés Cisneros, Michael J. Schnieders, Nohad Gresh, Yvon Maday, Pengyu Y. Ren, Jay W. Ponder, Jean-Philip Piquemal

**Affiliations:** a Sorbonne Université , Institut des Sciences du Calcul et des Données , Paris , France; b Sorbonne Université , Institut Parisien de Chimie Physique et Théorique , CNRS , FR 2622 , Paris , France; c Sorbonne Université , Laboratoire de Chimie Théorique , UMR 7616 , CNRS , Paris , France . Email: jpp@lct.jussieu.fr; d Universita di Pisa , Dipartimento di Chimica e Chimica Industriale , Pisa , Italy; e MATHCCES , Department of Mathematics , RWTH Aachen University , Aachen , Germany; f The University of Texas at Austin , Department of Biomedical Engineering , TX , USA; g Department of Chemistry , Wayne State University , Detroit , MI 48202 , USA; h Department of Chemistry , University of North Texas , Denton , TX 76202 , USA; i The University of Iowa , Department of Biomedical Engineering , Iowa City , IA , USA; j Sorbonne Université , Laboratoire Jacques-Louis Lions , UMR 7598 , CNRS , Paris , France; k Institut Universitaire de France , Paris , France; l Brown University , Division of Applied Maths , Providence , RI , USA; m Washington University in Saint Louis , Department of Chemistry , Saint Louis , MI , USA

## Abstract

We present Tinker-HP, a massively MPI parallel package dedicated to classical molecular dynamics (MD) and to multiscale simulations, using advanced polarizable force fields (PFF) encompassing distributed multipoles electrostatics. Tinker-HP is an evolution of the popular Tinker package code that conserves its simplicity of use and its reference double precision implementation for CPUs. Grounded on interdisciplinary efforts with applied mathematics, Tinker-HP allows for long polarizable MD simulations on large systems up to millions of atoms. We detail in the paper the newly developed extension of massively parallel 3D spatial decomposition to point dipole polarizable models as well as their coupling to efficient Krylov iterative and non-iterative polarization solvers. The design of the code allows the use of various computer systems ranging from laboratory workstations to modern petascale supercomputers with thousands of cores. Tinker-HP proposes therefore the first high-performance scalable CPU computing environment for the development of next generation point dipole PFFs and for production simulations. Strategies linking Tinker-HP to Quantum Mechanics (QM) in the framework of multiscale polarizable self-consistent QM/MD simulations are also provided. The possibilities, performances and scalability of the software are demonstrated *via* benchmarks calculations using the polarizable AMOEBA force field on systems ranging from large water boxes of increasing size and ionic liquids to (very) large biosystems encompassing several proteins as well as the complete satellite tobacco mosaic virus and ribosome structures. For small systems, Tinker-HP appears to be competitive with the Tinker-OpenMM GPU implementation of Tinker. As the system size grows, Tinker-HP remains operational thanks to its access to distributed memory and takes advantage of its new algorithmic enabling for stable long timescale polarizable simulations. Overall, a several thousand-fold acceleration over a single-core computation is observed for the largest systems. The extension of the present CPU implementation of Tinker-HP to other computational platforms is discussed.

## Abstract 2

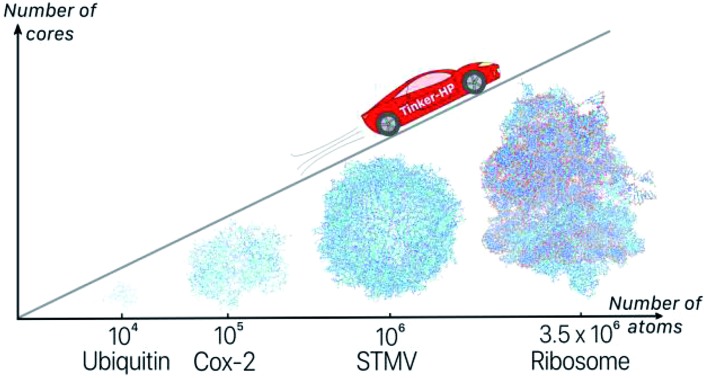
Tinker-HP is massively parallel software dedicated to polarizable molecular dynamics.

## 1 Introduction

Over the last 60 years, classical Molecular Dynamics (MD) has been an intense field of research with a high rate growth. Indeed, solving Newton equations of motion to resolve the time-dependent dynamics of atoms within large molecules allows to perform simulations in various fields of research ranging from materials to biophysics and chemistry. For example, in the community of protein simulations, classical force fields (FF) such as CHARMM,^1^ AMBER,^2^ OPLS,^3^ GROMOS^4^ and others,^5^ enabled large scale simulations on complex systems thanks to the low computational cost of their energy function. In that context, various simulation packages appeared, often associated to these FF such as the popular CHARMM,^6^ GROMOS^7^ and AMBER softwares.^8^ Among these, Tinker (presently version 8 (ref. 9)) was introduced in 1990 with the philosophy of being both user friendly and to provide a reference toolbox for developers. Later on, the evolution of computer systems enabled the emergence of massively parallel softwares dedicated to molecular simulations such as LAMMPS,^10^ NAMD,^11^ Gromacs,^12^ AMBER (PME-MD),^13^ DLPOLY,^14^ Genesis^15^ or Desmond.^16^ As they were granted the use of large computational resources, access to million atoms systems and biological time scales became possible.^17^ Nevertheless, up to now, such simulations are mainly limited to first-generation molecular mechanics (MM) models that remains confined to a lower resolution approximation of the true quantum mechanical Born–Oppenheimer potential energy surfaces (PES). However, beside these methods, more advanced second generation “polarizable” force fields (PFF) emerged in the last 30 years.^18^–^28^ Grounded on Quantum Mechanics (QM) and usually calibrated on the basis of Energy Decomposition Analysis (EDA),^29^ they go beyond pairwise approximation by including explicit many-body induction effects such as polarization and in some cases charge-transfer. Fluctuating charges, classical Drude approaches or point dipole coupled to point-charge models using distributed polarizabilities are among the most studied techniques aiming to include polarization effects.^28^ On the accuracy side, some PFF go beyond the point charge approximation incorporating a more detailed representation of the permanent and induced charge distributions using QM-derived distributed multipoles and polarizabilities.^18^,^19^,^24^,^26^ Recently, a third-generation PFF using distributed frozen electronic densities in order to incorporate short-range quantum effects^30^ appeared. In term of PES, these advanced force fields clearly tend to offer improved accuracy, better transferability and therefore are hoped to be more predictive. Unfortunately, everything has a cost: such elegant methods are more complex by design, and are therefore computationally challenging. Until recently the more advanced point dipole polarizable approaches were thought to be doomed for the simulation of realistic systems due to the evaluation cost of the polarization energy. Large scale polarizable production MD simulations were limited to the use of the Drude-type/point-charge model (using an extended Lagrangian propagation scheme)^31^ that was found to be more tractable than point dipole models (using iterative solvers) coupled to multipolar representation of the permanent charge distribution. Nevertheless, despite this scalability issue, time was not lost and accurate models were developed such as the Tinker package, original home of the multipolar AMOEBA PFF,^24^ specialized in offering a dedicated development platform with all required advanced algorithms for these accurate techniques. Moreover, ten years ago, a hardware technical revolution in the field of High Performance Computing (HPC), had a profound impact on MD simulations with classical FF.^32^ Indeed, the introduction of Graphical Processing Units (GPUs) offered a brute force hardware acceleration to MD packages thanks to simple- or mixed-precision implementations.^33^ Tinker benefited from the availability of this low cost but powerful type of hardware. It led to a GPU version of the code denoted Tinker-OpenMM.^34^ The code is based both on Tinker and on the OpenMM library (now version 7 (ref. 35)) which pioneered the use of GPUs with polarizable force fields. Tinker-OpenMM offers a 200-fold acceleration compared to a regular single core CPU computation giving access to accurate free energy simulations. However, when one considers the need for biophysical simulations, this acceleration remains not sufficient.

The purpose of the present work is to push the scalability improvements of Tinker through new algorithms to explore strategies enabling a 1000-fold and more speedup. These new developments aim towards modern “big Iron” petascale supercomputers using distributed memory and the code design also offers consequent speedups on laboratory clusters and on multicore desktop stations. The philosophy here is to build a highly scalable double precision code, fully compatible and consistent with the canonical reference Tinker and Tinker-OpenMM codes. As the new code remains a part of the Tinker package, it is designed to keep its user-friendliness offered to both developers and users but also to provide an extended access to larger scale/longer timescale MD simulations on any type of CPU platforms. The incentive to produce such a reference double precision code is guided by the will to also perform scalable hybrid QM/MM MD simulations where rounding errors must be eliminated. This will bring us not to cut any corners in our numerical implementation with the key mantra that one should not scale at any cost, as the algorithms developed in this interdisciplinary project should be based on solid mathematical grounds.

The paper is organized as follows. First, we will present the newly developed extension of 3D spatial decomposition and memory distribution to polarizable point dipole models that is at the heart of Tinker-HP for short-range interactions. Then we will detail the handling of long-range electrostatic and polarization interactions with a new framework coupling Smooth Particle Ewald to Krylov iterative and non iterative polarization solvers. We will then introduce the possibilities of the software and show benchmarks for selected applications in the context of the AMOEBA PFF.^24^,^36^ Finally, we will present functionalities of Tinker-HP that go beyond MD simulations in periodic boundary conditions as we conclude by drawing some perspectives about evolutions of the code towards next HPC platforms.

## 2 Accelerating polarizable molecular dynamics using massively parallel 3D spatial decomposition

In this section, we describe the first extension of 3D spatial decomposition to polarizable point dipoles models dedicated to production simulations. Indeed, in the past, point dipole model implementations in parallel have been limited to the use of a few dozen processors.^37^ In this section, we detail the parallelization strategy used in Tinker-HP to overcome this problem and to deal with local interactions, including the direct-space part of electrostatic and polarization interactions. The long-range, reciprocal field part of such interactions, is discussed in Section 3.

### 2.1 State of the art in massively parallel implementation of classical molecular dynamics simulations

**Fig. 1:**
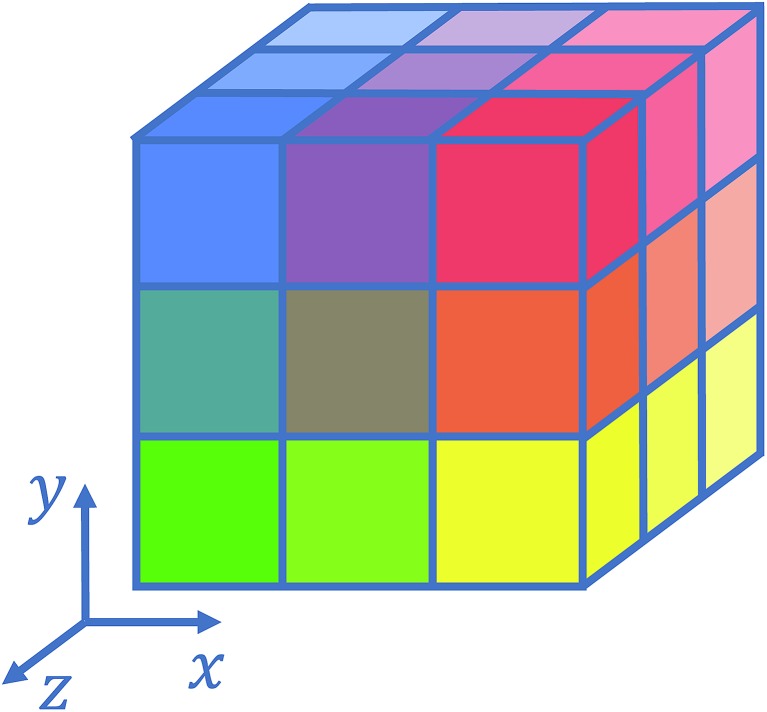
Example of 3D spatial decomposition.

Several strategies^10^–^12^,^16^ have been devised in order to treat short-range interactions on large-scale parallel computers using distributed memory parallelism. In Tinker-HP, we have implemented a spatial decomposition (or domain decomposition) method. In this approach, the simulation domain is decomposed in 3D blocks and each block is assigned to a processor. Each processor then handles the computation of the forces and the update of the coordinates for the atoms assigned to the block at each time-step. This strategy is motivated by the fact that the interactions considered are short-range, and that the positions of the atoms do not change much between two consecutive time-steps. An example of such a decomposition with 3 × 3 × 3 = 27 blocks is given in Fig. 1. One can show^10^ that if the cutoff (*r*_c_) involved in the short-range interactions is superior to the size of an edge of a block, which is the case with a high number of processors, the amount of data to be communicated in and out of each processor at each time step (the so-called communication volume) scales like 
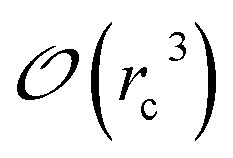
 (if the density of the system is uniform) independently of the number of processors. As a consequence, the communications are local which is an advantage of this method over the other ones. However, achieving a good load-balancing is harder using this strategy when the density of the system is not uniform or when the simulation box is not a parallelepiped.

Let us give more details about the algorithm and the main steps required to perform a time step of MD using this method. We assume that the simulated system resides in a box that has been divided in as many 3D blocks as the number of processors used. Let us focus on a processor that has been assigned a 3D block and let us assume that this processor knows the current positions, velocities and accelerations of the atoms currently belonging to this block. In integrator schemes such as velocity Verlet, the first integration step consists of an update of the local positions and a first update of the velocities. Because of these position changes, some atoms may cross the local block boundaries and need to be reassigned to neighboring blocks. This step, that we will call “reassign step” only requires local communications between a small number of neighboring processes.

In the second step, the forces are computed and used for the second update of the velocities. This requires the processor to know the positions of all atoms within the interaction cutoff, that have to be communicated from the processors assigned to the blocks that are at distance inferior or equal to the cutoff. We will call this step, which also involves local communications (but that may involve more distant processors than the previous one) “position comm” step. Once this is done, the algorithm loops back to the first step.

**Fig. 2:**
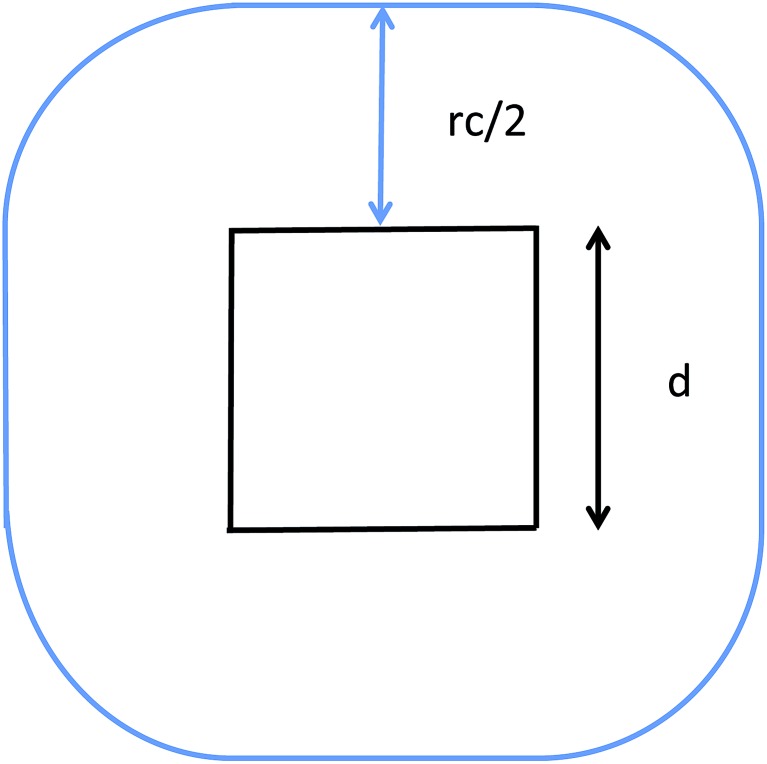
Illustration of the midpoint rule in 2D: the square of edge *d* represents a subdomain assigned to a process and the blue line delimits the area that has to be imported by the latter.

The communication volume involved in the position comm step can be reduced by taking into account the pairwise nature of the fundamental operations needed to compute the forces. Given a pair of atoms, in fact, one needs to choose which processor will perform the elementary force computation. This can be done on the basis of a geometrical argument. Among the various methods, that are also called neutral territory approaches,^38^ we choose the one presented by Shaw *et al.*,^16^ known as the midpoint method.^38^ This method picks out the processor that computes an interaction between two atoms as the one assigned to the subdomain where the center of the segment between the two atoms lies. As a consequence, each processor only needs to import information about atoms located at less than 
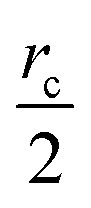
 from its block: one can show that the communication volume is then, with *d* being the size of an edge of a subdomain, 
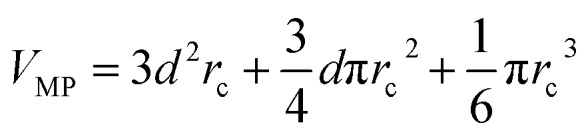
 as represented schematically in Fig. 2. This is a significant reduction with respect to the naive method,^38^ especially at a high processors count. Note, however, that within this scheme, a processor might need to compute the elementary interaction between atoms that do not belong to its block.

Furthermore, once the elementary pairwise computation has been done, we can take advantage of Newton's third law and communicate the force back to both processors from which the positions originated (“force comm” step). This additional communication cost is in general negligible compared to the computational gain represented by the reduction of the computations of the forces by half.

Additionally, the midpoint approach is simple enough not to complicate too much the implementation, which is ideal for a platform like Tinker-HP, meant to be shared with a community of developers. Nevertheless, more elaborate techniques are interesting and have been shown to reduce asymptotically the amount of data that need to be communicated in the “position comm” step and in the “forces comm” step. We are currently studying these methods in the context of PFF to compare them to the present midpoint approach. Some of them should appear in future releases of our code.

**Fig. 3:**
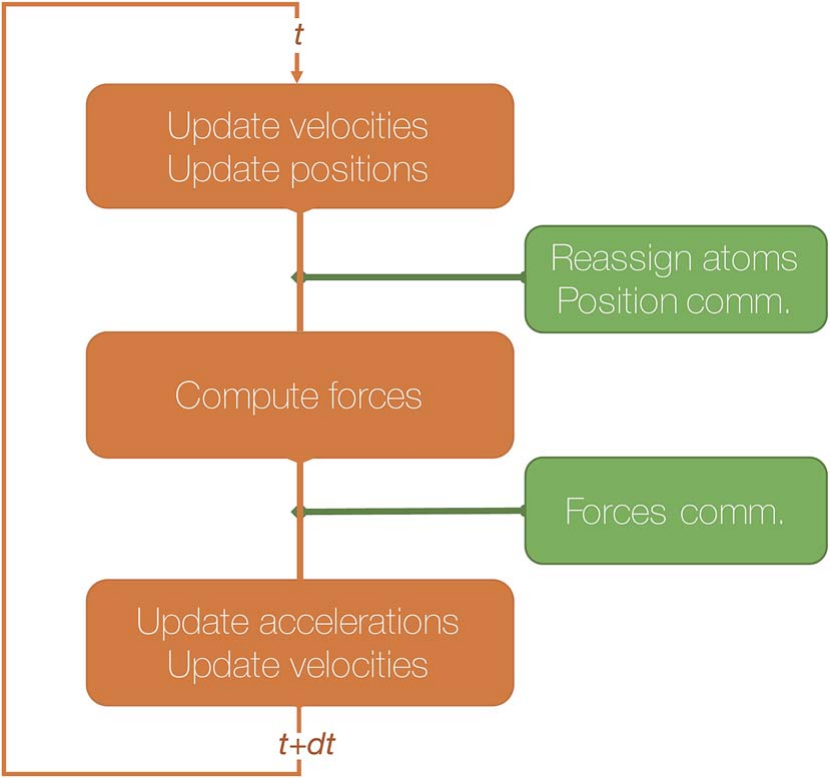
Schematic representation of a velocity Verlet step.

The algorithmic structure of a parallel (short-range) MD step with spatial decomposition is shown in Fig. 3.

To address load balancing issues that may appear in non-homogeneous systems (when equally sized subdomains contain a very different number of atoms), a procedure in which the size of the subdomains is iteratively changed has been implemented.

### 2.2 Distributed memory for simulations using point dipole models

Distributed memory parallelism allows one to scatter the memory among the processors and thus to run simulations that would not be possible because of memory limitations. In Tinker-HP, almost all data are distributed, this being possible by reallocation of dynamically allocated arrays at regular intervals. For example, during the computation of the non-bonded forces at a O(N) computational cost using the linked-cell method,^39^ the neighbor lists used, that are the most memory-consuming data structures of the program, are reallocated at the same frequency as they are updated. This is an important aspect allowing Tinker-HP to remain efficient on small computer clusters and desktop stations as the list builder will adapt to the situation.

Unfortunately, some data structures such as the arrays containing the global parameters are allocated once and for all and cannot be distributed. This is especially problematic for PFFs such as AMOEBA, that require more parameters than the classical ones: replicating these arrays for each processor would be prohibitive. This issue can be circumvented by using shared memory segments that can be managed with MPI (3.X) directives. This means that these data are allocated only once per node and are accessible by every processor within the node, reducing thus memory requirements by the number of processors of the node.

### 2.3 Adaptation of the 3D spatial decomposition to point dipole polarizable force fields

In this section, we will explain how the global concepts of 3D spatial decomposition can be adapted to the special case of the computation of the polarization energy and forces in PFFs. To our knowledge this is the first functional production implementation of such a technique in that context. Indeed, some of us proposed recently a 1D spatial decomposition^40^ implementation for AMOEBA. Here we propose a full extension to a 3D spatial decomposition to benefit from further performance enhancements. We will limit ourselves to the induced dipole model that is used in AMOEBA and that is the one implemented in Tinker-HP but the methodology is general and can be applied to various types of point dipole models.

The computation of the polarization energy in PFFs using the induced dipole formulation consists of two steps. First, a set of 3*N* (*N* being the number of polarizable sites) induced dipoles has to be computed by minimizing the functional
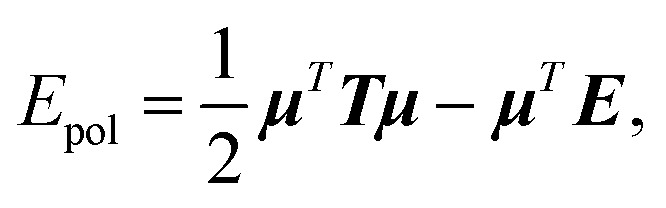
where ***E*** is a 3*N* vector representing the electric field produced by the permanent density of charge at the polarizable sites. This is equivalent to solving the 3*N* × 3*N* linear system

1 ***Tμ*** = ***E***,

where ***T*** is the polarization matrix. A detailed analysis of the polarization matrix and of the iterative methods that can be used to efficiently solve the linear system in eqn (1) can be found in ref. 41. Tinker-HP relies on Krylov approaches such as the Preconditioned Conjugate Gradient (PCG) and the Jacobi/Direct Inversion of the Iterative Subspace (JI/DIIS) algorithms. Their scalability and robustness have been discussed in previous works.^40^,^41^ Additionally, we recently introduced a powerful non-iterative Krylov solver with analytical derivatives named the Truncated Conjugate Gradient^42^,^43^ (TCG). Such a method has the same scalability as PCG but offers a reduced cost with conserved precision as it does not suffer from the typical drift observed in polarizable MD scheme based on iterative techniques. For all these iterative methods, the building blocks are matrix-vector products and scalar products. Focusing on the short-range, direct space part of the computation, each matrix vector product (MVP) is analogous to a force computation (as described in the previous section). Indeed, each MVP is analogous to computing a set of electric fields due to a set of dipoles so that in the context of a parallel MD with 3D spatial decomposition, communications of the “neighboring” dipoles are mandatory before each matrix-vector product: this is equivalent to the “position comm” step previously described. Since Newton's third law is used, symmetrical communications of some electric fields created by the local dipoles have to be communicated after the matrix-vector product computation: this is equivalent to the “forces comm” described above. The scalar products require a global reduction and are naturally distributed among the processors independently of the parallelization strategy.

The computation of the induced dipoles by iterative methods represents not only an important additional computational cost, but also an important communication cost, as at each iteration two of the three communication steps described in Section 2 are required.

**Fig. 4:**
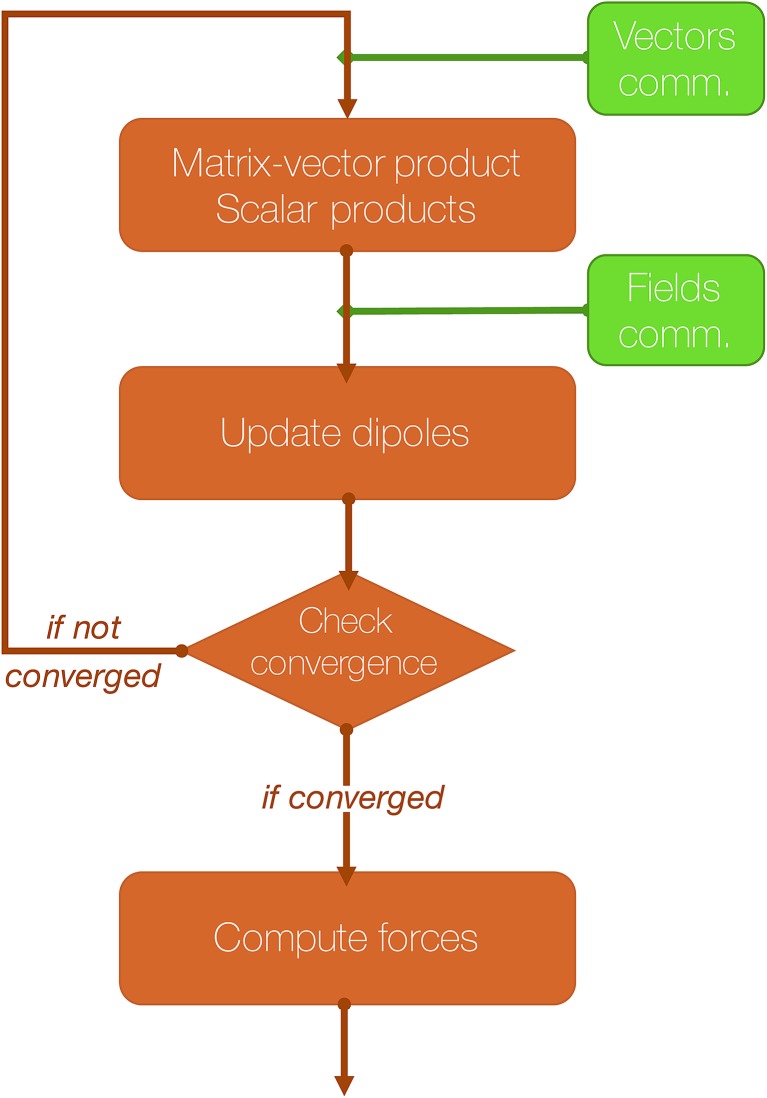
Schematic representation of an iteration of a polarization solver.

An essential part of our parallelization strategy is masking communication by computation in the parallel solvers whenever possible. This is achieved by using non-blocking MPI routines and by starting the receptions and the sendings of data as soon as possible, and, at the same time, verifying that the communications are finished as late as possible in the code, so that computations are being made between these two states. A schematic representation of a typical iteration of a polarization solver is shown in Fig. 4.

## 3 Parallel algorithm for point dipoles using smooth particle mesh Ewald

We present here new developments concerning the use of SPME (Smooth Particle Mesh Ewald) using distributed multipole electrostatics and polarizable point dipole models. Building on our previous work^40^ where we proposed a 1D decomposition of the distributed SPME grids, we now extend this formalism to the use of 2D pencil decomposition. Such an approach offers strongly improved performances especially when coupled to efficient iterative and non-iterative Krylov polarization solvers. In the previous section we focused the discussion on the parallelization strategy for short-range interactions. These include the bonded and van der Waals interactions, as well as the short range portion of the electrostatic and polarization interactions. The long-range part of such interactions needs to be handled separately, with a strategy that depends on the boundary conditions used for the simulation. Two main strategies exist in this regard: explicit solvent in periodic boundary conditions (PBC) and implicit solvation models. In this section, we focus on PBC. The additional possibility offered by Tinker-HP of treating the boundary with a polarizable continuum solvation model, namely, the Conductor-like Screening Model^44^–^46^ (COSMO), is presented in Section 6.

As we stated before, the method that we adopt for PBC is the Smooth Particle-Mesh Ewald^47^ (SPME). It has become a standard algorithm in modern biomolecular simulations to compute electrostatic interactions in periodic boundary conditions, thanks to its advantageous 
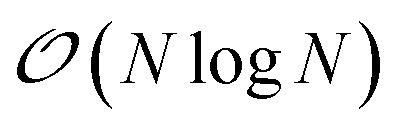
 scaling. The method has been extended to PFFs^48^ as well as to multipolar interactions,^49^ possibly including penetration effects.^50^

Let us explain the steps that are followed during a SPME computation for the electrostatic potential produced by distributed multipoles. The exact same considerations apply to the computation of the electrostatic and polarization forces and during a MVP computation during the iterative solution of the polarization equations. The electrostatic interactions are divided into two parts, one of which is short-range and is treated in the direct space, while the other is long-range and is treated in Fourier space. For the first, short-range part, the consideration made in Section 2 apply: we focus here on the reciprocal space computation. Such a computation requires the definition of a 3D grid and the use of Fast Fourier Transforms, which requires a significantly different parallelization strategy. The most standard one uses a 1D or 2D decomposition of the 3D grid and has been described elsewhere^12^,^40^ in detail. Let us summarize its main steps and analyze the parallelization strategy employed in Tinker-HP.

The SPME computation requires to distribute the multipoles on the grid using a B-spline interpolation and then to solve Poisson's equation in the reciprocal space. The distribution of the 3D grid is therefore what drives the parallelization strategy. In Tinker-HP, the grid is decomposed into 2D pencils, and each pencil is assigned to a processor. The first step of SPME consists into assigning charges or higher order multipoles to the gridpoints. As explained in our previous work,^40^ this operation requires local communications between processors assigned to neighboring portions of the grid.

The second step consist into switching to Fourier space by applying a forward FFT to the grid that has just been filled. In Tinker-HP, this is entirely handled by the 2DECOMP&FFT library.^51^,^52^

Then, the convolution with a pair potential^47^ is done in Fourier space, which is a simple multiplication that is naturally distributed among the processors without any necessary communication.

Finally, the result of this multiplication is transformed back to real space by applying a backward FFT, which is also taken care of by 2DECOMP&FFT in Tinker-HP.

**Fig. 5:**
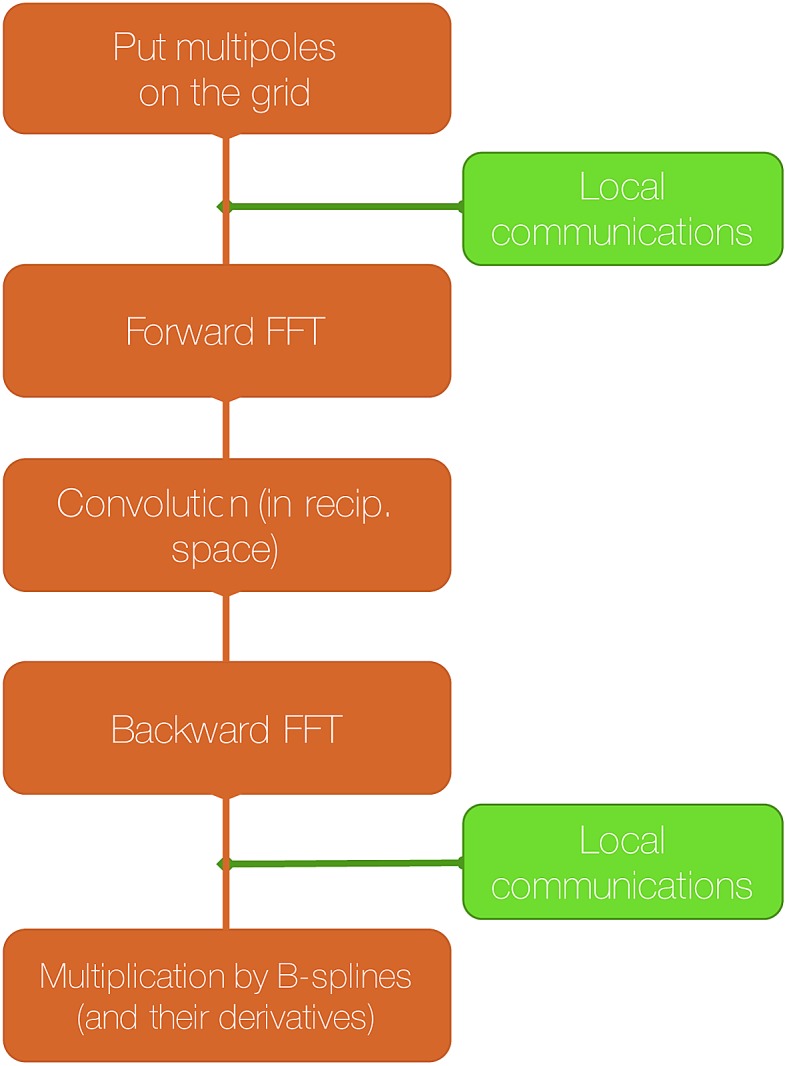
Schematic representation of the computation of the reciprocal part of the electrostatic energy and forces with SPME.

A final local set of communications between processors responsible for neighboring portions of the grid is done, followed by local multiplication with B-splines. A schematic representation of these steps is shown in Fig. 5.

Naturally, because the Fourier space decomposition of the grid may not fit exactly the 3D spatial decomposition, additional communications of positions are required before starting the reciprocal part of a SPME computation. Furthermore, when electrostatic or polarization forces are computed in this way, or after a matrix-vector multiplication in an iteration of a polarization solver, communication of some of these forces or dipoles are required.

Lagardère *et al.* showed^40^ that the reciprocal part of SPME presented just above does not scale as well as the direct part with the number of processors, because of the relatively poor parallel scaling of the FFTs. Furthermore, because reciprocal space and direct space computations are independent and because reciprocal space is usually computationally cheaper, a usual strategy is to assign a smaller group of processors to reciprocal space and the rest to the direct space. This strategy can be used in Tinker-HP for both permanent electrostatics and polarization.

In that case, a difficulty arises in PFF computations. The load balancing between direct and reciprocal space computations is in fact essential to achieve a good scalability. However, the relative cost of direct and reciprocal computations is different for permanent electrostatics and MVP required for the computation of the induced dipoles. At this moment, only heuristic strategies have been implemented in Tinker-HP to handle this problem.

## 4 Software possibilities

Tinker-HP is part of the Tinker 8 package and consequently it is fully compatible with the canonical Tinker and the Tinker-OpenMM (GPU) codes. Therefore, all Tinker's analysis and visualization tools are available with Tinker-HP. Details about these possibilities are not described here and can be accessed on the Tinker community website (http://tinkertools.org). The Tinker-HP source code is freely available to the academic community: details and downloading informations can be found on the Tinker-HP website (; http://www.ip2ct.upmc.fr/tinkerHP). In the following section, we detail the possibilities of the code that will be contained in the incoming public releases.

### 4.1 Polarizable molecular dynamics engine features

#### List builder

As we stated in the method section, Tinker-HP is designed to be used on all types of CPU-based computer systems ranging from desktop computer to supercomputers. To do so, the package embodies a fast O(N) massively parallel list builder that is designed for both an extensive use of a large number of cores and to enable also an efficient treatment on a small number of cores.

#### Polarization solvers

Massively parallel implementation of various polarization Krylov solvers are present and includes iterative methods such as PCG, JI/DIIS. Both approaches can be used in connection with Kolafaś Alway Stable Predictor (ASPC)^53^ that reduces significantly the iteration numbers for 1 fs and 2 fs timesteps simulations (see ref. 43 for discussion). An efficient non-iterative/fixed cost approach is also available: the Truncated Conjugate Gradient (TCG). TCG is implemented at the TCG1 and TCG2 levels with various refinements.^42^,^43^ The TCG approaches are a strong asset of Tinker-HP as they accurately reproduce energy surfaces at a reduced computational cost and provide analytical forces. Such an approach avoids numerical drifts appearing with iterative methods and therefore brings enhanced energy conservation for long simulations. It is also fully time-reversible and compatible with the use of larger time-steps.

It is important to point out that an important choice in the Tinker-HP strategy is to keep accuracy to the maximum by retaining a double-precision approach. By definition, GPUs have the strong advantage of using mixed precision which has been shown to produce more stability than simple precision computations. The strategy here is to build on the availability of the double precision to use algorithms that should/could not be used in mixed precision but are expected to be fully operational and faster in our case. For example, any CG methods are sensitive to precision (the symmetry of matrices being lost) as is the case for predictor-correctors such as the ASPC. Tinker-HP offers a full use of these strategies and compensates for the extra computational cost of double precision by more dependable algorithms.

#### Integrators

Most of the integrators available in Tinker have been implemented including, namely velocity Verlet, Beeman and RESPA^54^ which allows production MD simulations with 2 fs time steps, and 3 fs timesteps using H mass repartitioning.

#### Simulation ensembles and associated tools

NVE, NVT and NPT simulations are possible. Bussi and Berendsen thermostats are available. NPT simulations are also implemented with a Berendsen barostat.

#### Restraints and soft cores van der Waals

Position, distance, angle, torsions and centroid based harmonic restraints as well as softcore van der Waals and scaled electrostatics for free energy calculations are available.

#### Geometry optimization

To prepare large systems encompassing millions of atoms through geometry optimization, Tinker-HP offers a massively parallel version of Tinker's limited memory BFGS quasi-newton nonlinear optimization routine (LBFGS).

### 4.2 Available force fields

#### Advanced point dipole polarizable force fields

Tinker-HP allows for electrostatics to range from point charges to fully distributed multipoles (up to quadrupoles), point dipole polarization approaches using distributed polarizabilities^41^ coupled to Thole (or dual Thole) damping approaches as well as van der Waals interactions using the Lennard-Jones or the Halgren functions. This choice was motivated as these functional forms have been extensively used by various research groups that could therefore easily use Tinker-HP with their own parametrizations. Presently, two polarizable force field models, both relying on the Thole/point dipole polarization model, are available. The first model is the AMBER f99 polarizable model. It is limited to point charges to compute the permanent electrostatics and uses a 6–12 Lennard Jones for the van der Waals.^20^,^55^ The second is the AMOEBA polarizable model which has been shown to have a wide applicability for systems ranging from liquids to metals ions, including heavy ones, in solution and to proteins and to DNA/RNA.^24^,^36^,^37^,^56^–^58^ A major difference compared to the AMBER model is the replacement of the fixed partial charge model with polarizable distributed atomic multipoles till quadrupoles moments, allowing accurate reproduction of molecular electrostatic potentials, and higher resolution rendering of difficult directional effects in hydrogen bonding and other interactions. van der Waals interactions are also different and use the Halgren buffered 14–7 function.^59^ The AMOEBA polarizable model responds to changing or heterogeneous molecular environments and its parameterization was performed against gas phase experimental data and high-level quantum mechanical results. The AMOEBA model includes high accuracy water model as well as parametrization for organic molecules, proteins,^60^ ions and DNA/RNA complexes.

#### Classical force fields

By construction, the software is able to perform classical force field simulations following the canonical Tinker initial implementation of the AMBER, CHARMM and OPLS potentials. Such force fields also benefit from the speed up of the massively parallel framework but our objective is to reach comparable performance to the AMBER and CHARMM (Domdec^61^) CPU implementations. The detailed analysis of such code capabilities being beyond the scope of this paper, fully dedicated to polarizable models, and it will be discussed elsewhere. However, it can be noted that classical MM that requires much less work than PFFs allows for a 5–8 acceleration of the production per day over AMOEBA (depending on the use of TCG *vs.* PCG solvers) on the same computational platform, and will be used for hybrid simulations with PFFs coupled to non-polarizable parts of the system. For higher performances using Tinker, one could use the Tinker-OpenMM access to the OpenMM library implementation of such classical FF. For example, it is possible to produce 305 ns per day for DHFR with the same GTX 1080 card (mixed precision) and settings used in this work using the AMBER force field.

## 5 Benchmarks and applications using the AMOEBA polarizable force field

The present implementation has been extensively tested and reaches exactly the same accuracy as the canonical Tinker for polarizable force field when considering analogous algorithms, allowing Tinker-HP to produce reference computations. All the proposed benchmarks use the AMOEBA force field. We tested the performances of Tinker-HP on various systems. We studied the scalability of the code dealing with homogeneous systems such as bulk water, and inhomogeneous systems ranging from ionic liquids to proteins. Finally we tested our approach on very large biosystems.

### 5.1 Computer platforms

All tests have been performed on the Occigen machine at GENCI (CINES, Montpellier, France) and at CYFRONET (Krakow, Poland) on the Prometheus machine. Occigen is a Bullx DLC with Intel Xeon E5-2690 v3 (24 Haswell cores at 2.6 GHz per node) and Intel Xeon E5-2690 v4 (28 Broadwell cores at 2.6 GHz per node), Infiniband FDR and 128 Go of memory per node. Prometheus is a HP Apollo 8000 with Intel Xeon E5-2680 v3 (24 Haswell cores at 2.5 GHz per node), Infiniband and 128 Gb of memory per node. For consistency, all results are given for Haswell processors. We observed an average four per cent gain in speed on the Broadwell configuration, especially for a suboptimal number of cores, *i.e.* before the scaling limit. Some timings have been obtained using Tinker-OpenMM on GPU cards (NVIDIA GTX 970 and GTX 1080), the best GPU results (GTX 1080) can be found in Table 3 below, the GTX 970 productions being roughly half of the GTX 1080 ones.

### 5.2 Simulations setup

Benchmark simulations (except free energies) were made in the NVT ensemble with a Verlet/RESPA multi-time step integrator with either a 2 fs or a 3 fs time-step (using hydrogen mass re-partitioning in the latter case) for the non-bonded forces and half of this value for the bonded forces. Two Krylov solvers were considered here: iterative PCG and non-iterative TPCG, both using a diagonal preconditioner.^41^,^42^ Note that we report here the first results ever using TCG coupled to SPME. The convergence criterion for the PCG iterative solver was set to 10^–5^ D. Electrostatics and polarization interactions were treated using the PME algorithm with a real space Ewald cutoff of 7.0 Å. The van der Waals cutoff was set 9.0 Å without any long-range correction.

### 5.3 Homogeneous systems: water boxes and ionic liquids

#### Water boxes

We first benchmarked the code on cubic water boxes of increasing size: from 96 000 atoms up to 23.3 millions atoms. Table 1 summarizes the characteristics of these boxes: their size in Angstroms, the number of atoms they contain, the size of the associated PME grid and the name with which they will be referenced in the rest of the paper.

**Table 1:** Water boxes used for benchmark purposes

System	Puddle	Pond	Lake	Sea	Ocean
Number of atoms	96 000	288 000	8 640 000	7 776 000	23 328 000
Size (of an edge) in Angstroms	98.5	145	205.19	426.82	615.57
Size (of an edge) of the PME grid	120	144	250	432	648

**Fig. 6:**
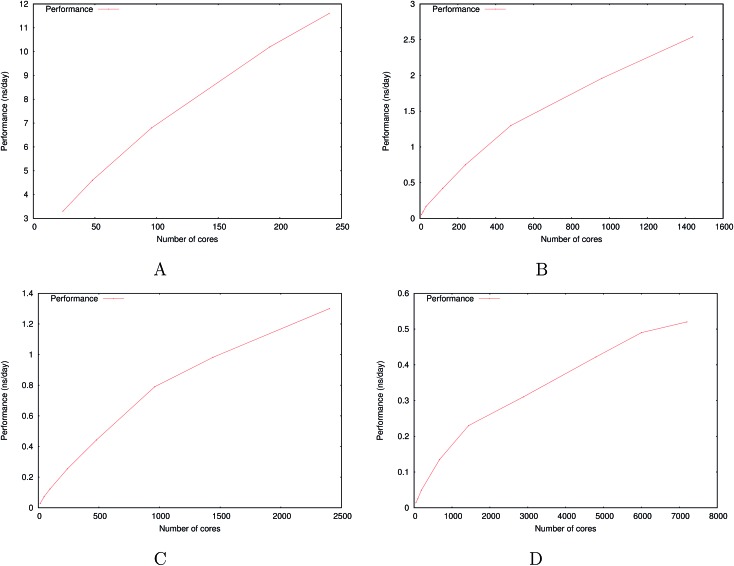
Performance gain for the [dmim^+^][cl^–^] ionic liquid system (A) and the Puddle (B), Pond (C) and Lake (D) water boxes.

Fig. 6 show the detailed scalability up to almost 1 million atoms.

**Fig. 7:**
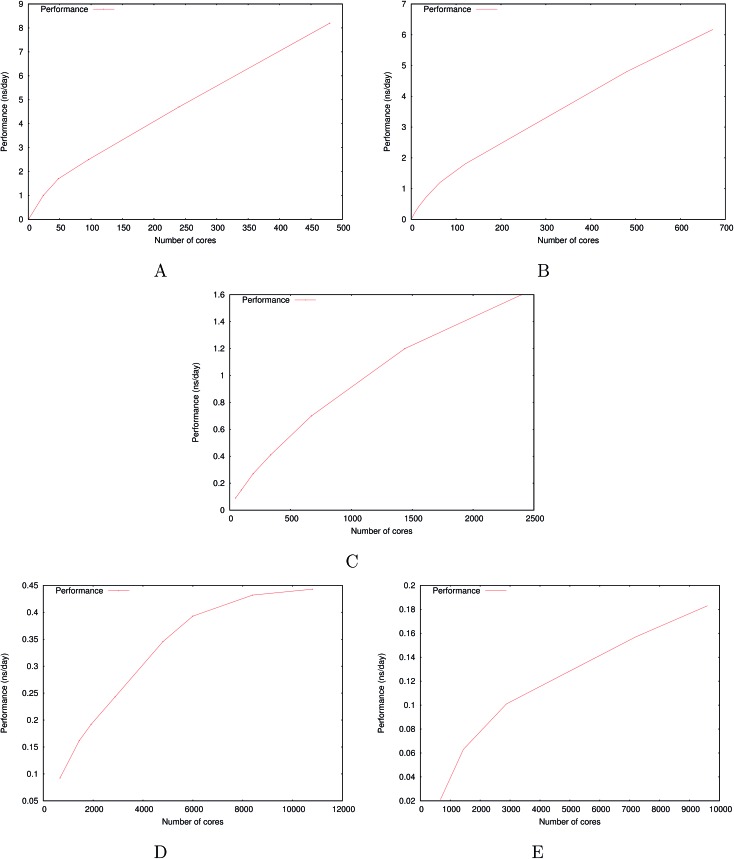
Performance gain for the ubiquitin protein in water (A), the dihydrofolate reductase protein (dhfr) in water (B), the COX-2 system in water (C), the satellite tobacco mosaic virus in water (D) and the ribosome in water (E).

A very good scalability is observed in the three cases. Table 3 displays the best production timings in ns per day. The code appears to be competitive with the GPU numbers extracted from Tinker-OpenMM even for a system such as the smallest waterbox test (Puddle, 96 000 atoms). In this case, Tinker-HP is already 1.5 faster than a GTX 1080 card (3 times for a GTX 970) but with double precision compared to mixed precision arithmetics used by GPUS. As we will discuss later in the case of proteins, the newly introduced 3D domain decomposition algorithmic for polarizable FF becomes more beneficial when the size of the system grows and a first advantage of Tinker-HP is to be able to use the distributed memory system of the CPU platform. Also for such large systems numerical instabilities of the polarization solvers that result in energy drifts^40^–^43^ are a key error that must be contained. Double precision is highly preferable when one wants to use advanced conjugate gradient solvers (and Krylov approaches in general). Tinker-HP has an advantage as it affords mathematically robust solutions for “drift-free” polarization solvers (Truncated Conjugate Gradient, TCG^42^,^43^) with analytic forces. Such techniques allow for (very) long simulations. A stable adaptation of these methods to mixed precision hardware (*i.e.* GPUs) is underway but is mathematically non-trivial. Note that for short to medium simulations of a few dozen ns, the discussion is without object as the drifting issue will remain negligeable offering a full applicability of GPUs acceleration. However, towards and beyond the microsecond, the analytical forces polarization solvers will be key for stable polarizable simulations. For the other benchmark cases, the speedup increases to a 5 and 6-fold over a GTX970 (2 and 3-fold over a GTX1080) for 288 000 atoms (Pond) and 864 000 atoms (Lake) water boxes respectively. For the Lake box, a detailed analysis of the scaling against ideal scaling is provided in ESI S2.† We then pushed the code towards its limits by testing very large systems including 7 776 000 and 23 300 000 atoms respectively. At these levels, GPUs have memory limitations that makes such simulations impossible, which is not the case with supercomputers relying on distributed memory. These “computational experiments” took place on the Prometheus supercomputer (CYFRONET, Krakow, Poland) and enabled us to test for the validity of the code on a very large scale. Results show that Tinker-HP is still operational beyond 20 million atoms. Of course, the production really slows down to a few dozen ps per day but the performance is noticeable as it would be quite enough to compute properties such as electrostatic potentials or even a short ns-scale molecular dynamics. Thus, one can expect, depending on the machine used, to produce a ns in a few weeks on the largest Ocean water box using TCG2/RESPA (3 fs). It is worth noticing that the largest computation was limited only by the computer system availability and that presently larger systems are potentially accessible with more computational resources. However, such very large computations require a different setup than the others due to memory limitations and communication issues. Indeed, for such a large number of atoms, FFTs really become severely time limiting and intra-node communications strongly affect performances. One solution that was used for Ocean was to only use a fraction of the cores of a node to take advantage of the node memory without suffering from excessive communications. That way, if the Ocean test ran on 12 288 cores on 512 nodes, we used only 6 cores/node (on 24) to actually perform the computation. This gave us the possibility to better use the bandwidth of the interconnect cards (by reducing contention in MPI transfers between cores and cards), a strategy that compensates for the lack of active cores and that can be used for any system size. We used the same strategy to a lower extent for Sea as 17 cores out of 24 were active. Overall, a rough estimate for the fastest Broadwell CPU configuration (Occigen) is that using a RESPA (3 fs)/TCG2 setup, a routine production of 1 ns per day is reachable for a million atoms. Such a value is a combination of various hardware setups that are not only dependent on the CPU speed (and numbers), as the interconnection cards have a strong influence on the final results (Fig. 7).

#### Ionic liquids

Room temperature ionic liquids (ILs) are molten salts at room temperature that are formed by the combination of organic or inorganic cations with (generally) inorganic anions. These compounds exhibit a wide variety of useful properties that led to their use in numerous applications.^62^–^65^ The unusual properties observed in ILs arise from the inter- and intra-molecular interactions of the constituent ions. Thus, the computational simulations of these systems greatly benefit from the use of highly accurate potentials. Recently AMOEBA parameters for several ILs have been developed and applied for various systems.^66^–^68^ It is known that polarization effects result in better reproduction of transport properties.^69^–^72^ In addition, ILs are viscous fluids and it is thus necessary to perform relatively long MD simulations. Therefore, Tinker-HP is an ideal platform for these systems given its HPC capabilities and implementation of significantly more accurate and efficient algorithms for the evaluation of the polarization component. Indeed, ILs usually require a lot more iterations than standard molecules with standard solvers such as JOR (Jacobi Over Relaxation, see ref. 41), which is not the case with Krylov solvers such as PCG or TCG, with which such systems have been tested.^42^ As a first example, simulations were performed for 1,3-dimethylimidazolium imidazolium/chloride ([dmim^+^][cl^–^]) for 200 ns using the parameters reported by Starovoytov *et al.*^66^ The results calculated with Tinker-HP are in very good agreement with the previously reported results, with the added advantage that Tinker-HP provides excellent scaling, with production runs for a system of 216 ion pairs (in a cubic box of 35.275 Å, a PME grid of 48 × 48 × 48 and a 7 Å real space cutoff) of more than 11.5 ns per day on 240 cores. Therefore, Tinker-HP enables simulations of IL systems in the hundreds of ns up to μs timescales.

### 5.4 Speeding up free-energy computations: assessing large water box hydration free energies computations

The observed speed-up on water boxes led us to test the performance AND the accuracy of free energy computations using large water boxes to compare them to initial works using AMOEBA and the canonical Tinker software. The hydration free energies for water, benzene, K^+^ and Na^+^ were calculated by summing up the free energies of three thermodynamic steps, solute discharging in a vacuum, solute van der Waals coupling with solvent, and solute recharging in solvent. For K^+^ and Na^+^, since the standard state in simulation was 1 mol L^–1^ and the standard state in experiment was 1 atom, the free energy difference between the two states of 1.87 kcal mol^–1^ was added to the final results. The softcore van der Waals potential was used as in our latest work with Tinker. A total of 21 alchemical states were considered, and a 2 ns NVT simulation was performed at each state. The RESPA (2 fs) integrator was employed as the temperature was maintained at 298 K by the Bussi thermostat. The vdW interaction was truncated at 12.0 Å as SPME used a real-space cutoff of 8.0 Å and a 72 × 72 × 72 grid. The Bennet Acceptance Ratio (BAR)^73^ method was used to extract the free energies between states. In order to test the computation efficiency, the solute molecule was immersed in a large cubic simulation box of 6000 water molecules. The length of the box was 56 Å and 192 cores were used for each simulation with 48 dedicated cores for PME. This number of core is suboptimal but already provides a very good speedup as all windows were launched simultaneously on a total of 4032 cores, a computer resource that is commonly accessible in modern computer centers. Each total free energy evaluation took 18 hours to complete using a PCG coupled to Kolafa's predictor-corrector (ASPC) algorithm with a 10^–5^ convergence threshold. The hydration free energies for water, benzene, sodium and potassium are listed in the table of the ESI S1,† together with results from previous work. For all four solute molecules, there is excellent agreement between Tinker-HP and previous simulations using either BAR or OSRW (Orthogonal Space Random Walk) method.^74^ The values converge at 2 ns with a statistical error of around 0.1 kcal mol^–1^. The hydration free energies for potassium obtained from Tinker-HP and the Tinker published results are slightly different because the Tinker historical work did not use the softcore van der Waals potential at that time, but appears fully consistent with the present canonical Tinker result. Overall, Tinker-HP appears reliable and very efficient for the calculation of solvation free energies with huge gain in terms of computation time. Of course, further tests on more complex free energy computations are required to test all the possible combinations of TCG and RESPA algorithms. If TCG2 is really accurate and fast, TCG1 is significantly faster but these procedures have not been extensively tested yet and their evaluation concerning their applicability to free energy computations will be the subject of a larger study. In any case, TCG2 would lead to a computing time reduction of the same computations to roughly 14.5 hours and TCG1 to 12.5 hours. Such studies will benefit from the computational platform introduced in Tinker-OpenMM that allows computing absolute binding and relative alchemical approach as well as relative binding affinities of ligands to the same host. As an immediate other perspective, the OSRW results extracted from the canonical Tinker are presented in the table. This approach leads to very similar results to the BAR approach but requires up to 5 times less computer time. OSRW is currently under implementation in Tinker-HP. These results give an idea about the new possibilities offered by massive parallelism for free energies evaluations: the discussed simulations that initially took months are now possible within half a day and soon in a couple of hours with OSRW within Tinker-HP.

### 5.5 From proteins to realistic biosystems

To study the scalability and applicability of the Tinker-HP platform to complex non homogeneous systems, we tested various systems starting from the “small” ubiquitin protein (9737 atoms), and prototypic dihydrofolate reductase (dhfr, 23 558 atoms) which is the reference protein test case extracted from the joint AMBER/CHARMM benchmark (http://ambermd.org/amber10.bench1.html). We push the code towards the simulation of very large biosystems tackling the COX-2 dimer, the Satellite Tobacco Mosaic Virus (STMV) and the ribosome full structures in polarizable water. All timings are obtained for equilibrated systems.

The characteristics of the inhomogeneous systems simulations boxes used for benchmark are summed up in Table 2.

**Table 2:** Biosystems used for benchmark purposes

Systems	Ubiquitin	Dhfr	COX-2	STMV	Ribosome
Number of atoms	9732	23 558	174 219	1 066 228	3 484 755
Size (of an edge) in Angstroms	54.99 × 41.91 × 41.91	62.23	120	223	327.1
Size (of an edge) of the PME grid	72 × 54 × 54	64	128	270	360

#### Small proteins: ubiquitin and DHFR

We started our study by testing Tinker-HP on small proteins were 3D domain decomposition is expected not be fully efficient (our water boxe study started at 96 000 atoms, which is 4 times the size of DHFR and 10 times that of Ubiquitin). Surprisingly, results remain competitive with GPUs which are fully taking advantage of their computing power for such a range of systems with low memory requirements. DHFR allows to study in depth the code behavior in that system size range. Indeed, the best production time for a use of all cores of a node brings us to a 7.69 ns per day using TCG2. This production time is really close to the 8.29 ns per day exhibited by Tinker-OpenMM on a GTX1080 (see Table 3). If we used the same number of cores distributed on more nodes, to use the same technique we used on the large ocean and sea water boxes, the performance extends to 8.79 ns per day. These numbers make Tinker-HP very competitive for these small systems on a reasonable number of cores that is easily accessible on modern supercomputers. In addition, one can note that most of the recent machines use Broadwell Xeon that gives slightly better performances by a few percents. In other words, Tinker-HP is able to compensate for the computational burden of the use of double precision thanks to its new algorithmics compared to the accelerated mixed precision GPUs thus reaching both speed and accuracy. A detailed analysis of the DHFR scaling against ideal scaling is provided in ESI S2.† As one could expect, the deviation to the ideal scaling is higher than in the case of the previously larger Lake water box: larger the system is, closer to the ideal scaling we get.

**Table 3:** Best production time (ns per day) for the different test systems (AMOEBA force field) using various methods. Number of atoms and optimal number of cores are given for each systems. All timings are given for Intel Haswell processors. Reference canonical Tinker CPU times are given for Open-MP computations using 8 cores. All computations were performed using a RESPA (2 fs) integrator if not specified otherwise. ASPC = Always Stable Predictor Corrector.^53^ N.A. = Non Applicable due to memory limitations. GPU production times were obtained using the Tinker-OpenMM software^34^ (CUDA 7.5), the JI/DIIS solver and a GTX 1080 NVIDIA card

Systems	Ubiquitin	DHFR	COX-2	STMV	Ribosome
Number of atoms	9737	23 558	174 219	1 066 628	3 484 755
Tinker-HP number of CPU cores	480	680(960)	2400	10 800	10 800
PCG (10^–5^ D, ASPC)	8.4	6.3(7.2)	1.6	0.45	0.18
TPCG2	10.42	7.81(8.93)	1.98	0.56	0.22
TPCG2/RESPA (3 fs)	15.62	11.71(13.39)	2.98	0.84	0.34
CPU OPEN-MP	0.43	0.21	0.024	0.0007	N.A.
GPU (GTX 1080)	10.97	7.85	1.15	N.A.	N.A.

Systems (water boxes)	Puddle	Pond	Lake	Sea	Ocean
Number of atoms	96 000	288 000	864 000	7 776 000	23.3 × 10^6^
Tinker-HP number of CPU cores	1440	2400	7200	7104	12 288
PCG (10^–5^ D, ASPC)	2.54	1.3	0.52	0.062	0.0077
TPCG2	3.10	1.59	0.63	0.076	0.01
TPCG2/RESPA (3 fs)	4.65	2.38	0.95	0.11	0.014
CPU OPEN-MP	0.050	0.014	0.003	N.A.	N.A.
GPU (GTX 1080)	2.06	0.80	0.21	N.A.	N.A.

#### Larger systems: COX-2, STMV and ribosome solvated in water

For larger systems, as it was shown for the water boxes, the 3D domain decomposition speedup is taking full effect and the distributed memory approach offers an access to systems that were up to now restricted to classical non-polarizable force fields implemented in HPC packages. The benchmarks of Table 3 show that the discussion is fully transferable to non-homogeneous systems as realistic simulation times on a reasonable number of cores are reachable for the COX-2, STMV and ribosome systems allowing for meaningful simulations. The table displays a test for the COX-2 dimer (part of the Tinker benchmark suite, see ; https://dasher.wustl.edu/tinker/distribution/bench/) for which 1.6 ns per day are possible on 2400 cores, a computer resource that is easily accessible in supercomputer centers. If one wants to push the performances, one ns simulation can be achieved in a little more than a day on the STMV structure (taken from the NAMD website: ; http://www.ks.uiuc.edu/Research/namd/) which is not accessible to our GPU implementation due to memory requirements. Such a result is really extremely promising, considering that STMV encompasses more than a million atoms within the full virus structure including its full genetic materials, the whole system being fully solvated in water. Such simulations are indeed relatively recent even for classical force fields as the Schulten group only produced the first studies 10 years ago.^75^ The present extension of the simulation capabilities to advanced multipolar polarizable force fields opens new routes to the understanding of complex biosystems. Indeed, as we have seen, Tinker-HP is able to go far beyond the million atom scale and studies on the ribosome become possible following early studies (see ref. 76 and references therein). We built a model for benchmark purposes for the 70 s ribosome from *Thermus thermophilus* containing nearly 5000 nucleotides and over 20 proteins, with over 4100 sodium ions to neutralize the nucleic acid, and about a million water molecules for a total of 3 484 755 atoms. Presently, three days are necessary to produce a ns allowing for a very detailed study of such an important structure. We expect even free energy studies to be feasible. Various incoming studies will analyze more in-depth the use of PFFs to such mostly important biosystems.

## 6 Beyond classical MD simulations in periodic boundary conditions

So far, we have presented the capabilities of Tinker HP in the context of PBC classical molecular dynamics simulations. We have discussed the parallelization strategy and showed benchmark results that demonstrate the scalability and performances of the code. While Tinker-HP is mainly a molecular dynamics code, it is not limited to PBC classical simulations and can be used for different applications. In particular, Tinker-HP offers the possibility of performing non-periodic MD simulation with a polarizable force field such as AMOEBA using a polarizable continuum solvation model as a boundary. This possibility is not our main choice for MD simulation and, as a consequence, has not been as thoroughly optimized as the PBC code. Furthermore, it involves a few computational steps that scale quadratically with respect to the size of the system, making it not suitable for the very large systems presented in Section 5. However, the possibility of computing the energy and forces with non-periodic boundary conditions and with a continuum boundary opens the way for using Tinker-HP as a module to handle the classical part in a polarizable QM/MM(/continuum) calculations,^77^–^81^ including the computation of molecular properties and *ab initio* multiscale QM/MM MD simulations. These calculations are usually dominated in computational cost by the QM part, making the quadratic scaling of the classical part a minor issue. Nevertheless, the scalability of Tinker-HP paves the way to large-scale polarizable multiscale simulations.

In this section, we will describe the non-periodic code in Tinker-HP, based on the recently proposed ddCOSMO,^45^,^46^,^82^,^83^ a domain decomposition (dd) discretization of the conductor-like screening model.^44^ We will then discuss two complementary QM/MM strategies that can be used to couple Tinker-HP to a quantum-mechanical code.

### 6.1 Implicit solvent: ddCOSMO

Continuum solvation models^84^,^85^ (CSM) are a well-established technology in both quantum chemistry and MD. The CSM developed for MD are usually based on the Generalized Born (GB) Ansatz, or its multipolar generalization, which approximate the solution to the electrostatics equations in the presence of a continuum with an additive energy term. Methods developed in quantum chemistry rely, on the other hand, on a rigorous numerical solution of Poisson's equation. Such models are much more expensive than the GB counterpart; however, since these models have been developed for quantum mechanical calculations, and therefore for up to medium-sized systems, their computational cost is not a real limitation in QM calculations. Nevertheless, it has always prevented their application to MD simulations. The use of a polarizable CSM is of particular interest when a PFF is used due to the natural consistency between the two approaches. Recently, a new discretization to COSMO has been proposed. Such a new discretization, named ddCOSMO, has been developed when the molecular cavity is made of interlocking spheres (*i.e.*, van der Waals cavity) and has been extensively described elsewhere.^46^ The dd approach offers huge advantages since the matrix to be inverted to solve the model at each time step is highly sparse: as a consequence, the model scales naturally linearly with the size of the system and the iterative solution to the ddCOSMO equations is perfectly suited for a parallel implementation in which the spheres that constitute the cavity are distributed among cores.

The parallelization strategy adopted for the ddCOSMO implementation follows the spatial decomposition logic discussed in Section 2. Again, we divide the space occupied by the system into blocks and assign a block to each CPU. The CPU is then responsible for updating the positions, speeds and accelerations of the atoms belonging to it block. However, there are two important differences compared to the spatial decomposition discussed for short-range interactions. First, the space occupied by the solute is not a cube or a regular geometrical configuration but rather a cavity whose shape depends on the configuration of the solute. Second, the cavity is not fixed during the simulation as it evolves with the solute.

To address the first issue, we define the blocks by enclosing the solute in the smallest parallelepiped containing it and we divide this parallelepiped into smaller ones. This strategy presents the advantage of allowing us to reuse the whole machinery that has been described in Section 2. However, such a strategy can imply potential load balancing issues that require to be addressed, especially when a high number of processors is used. Again, an iterative procedure has been implemented to determine the optimal sizes of the sub-domains.

To solve the second issue, one should in principle recompute the enclosing parallelepiped at each time step. To avoid the cost of performing such an operation, we build a slightly larger parallelepiped and recompute its geometry only once every few MD steps (*n* = 20 for example).

In Tinker-HP, the solution to the ddCOSMO linear equations is computed by using the JI/DIIS iterative solver also used for the polarization eqn (1). The iterative procedure requires to compute MVP with the sparse ddCOSMO matrix, which can be done both very efficiently and involving only local communications. However, the right-hand side of the ddCOSMO equations depends on the electrostatic potential created by the solute's permanent and induced multipoles. In the current implementation, the potential is computed *via* a double loop, which implies a 
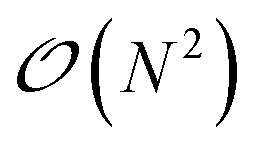
 computational cost. Furthermore, an “all to all” communication of the positions of the system is required prior to this computation.

Thus, the computational bottleneck in terms of both computational complexity and parallel efficiency lies in the computation of the right-hand side. If AMOEBA/ddCOSMO MD simulations have been shown to be possible,^46^ this kind of boundary is not competitive with SPME in term of pure polarizable MD production. However, as we stated at the beginning of this section, the advantage of the ddCOSMO implementation is to provide a boundary condition for multiscale simulations. In particular, having non-periodic boundary conditions is ideal when working with localized basis functions in QM computations.

Detailed benchmark results of the current parallel implementation are presented in ESI S3.†

### 6.2 Multiscale modeling and polarizable QM/MM

The PFF/ddCOSMO framework described in this section is a starting point for multiscale, polarizable QM/MM simulations. This is a fundamental direction for Tinker-HP as PFFs such as AMOEBA provide a high-quality embedding strategy for QM systems with various potential applications. For instance, in a recent publication, some of us showed how a DFT-based QM/AMOEBA description is able to model electronic excitations in aqueous solution^80^ for systems that interact in a specific and structured way with the environment. An *ab initio* QM/MM MD strategy has also been recently proposed.^81^

The present QM/MM possibilities of Tinker-HP follow two complementary strategies. Tinker-HP can be used as an external embedding tool, or can be directly coupled to a QM code in order to obtain a fully self-consistent polarizable QM/MM implementation.

The first strategy is the one followed in LICHEM^77^ (Layered Interacting CHEmical Model), that provides a QM/MM interface with unmodified quantum chemistry software suites such as Gaussian,^86^ PSI4,^87^ and NWChem^88^ to perform QM/MM calculations using the AMOEBA force field. This is done by approximating AMOEBA's multipolar distribution, with a set of point charges,^89^ which can then be read by the QM code. This choice is motivated by the idea of developing an interface with existing QM codes with non-native multipolar QM/MM capabilities. LICHEM extracts forces and energies from unmodified QM packages to perform a variety of calculations for non-bonded and bonded QM/MM systems, the latter by using the pseudobond formalism explicitly extended for QM/MM with PFFs.^77^,^90^,^91^ The calculations available in LICHEM include geometry and reaction path optimizations, single-point energy calculations, Monte Carlo, PIMD, *etc.*

Currently, the polarization component for the QM/MM interaction term in LICHEM is not fully self-consistent due to the use of unmodified QM codes. This is because only the field from the permanent multipoles from the MM subsystem is included in the effective Hamiltonian for the polarization component of the QM/MM interaction. However, as has been shown previously, this approximation, coupled with the fact that the QM and MM subsystem polarization is fully considered results into the recovery of over 80% of the total QM/MM self-consistent polarization.^77^,^92^

For the computation of electronic properties and full hybrid MD simulations, a second QM/MM approach can be pursued. This approach proposes a fully self-consistent treatment of the electronic density and the MM polarization and requires a modification of the QM self-consistent field routines. A QM/AMOEBA implementation that couples Tinker-HP to a locally modified version of the Gaussian suite of programs^86^ has been recently introduced.^80^,^81^ Such a strategy enables to use a DFT/AMOEBA based polarizable QM/MM strategy to compute the energy and response properties of an embedded system, as well as to perform Born–Oppenheimer (BO) hybrid QM/MM MD. The latter is accelerated through the use of an extended BO Lagrangian approach (XL-BO),^93^ which provides enhanced guess for the electronic density at every time step and allows for a stable hybrid MD with enhanced energy conservation.

In short, Tinker-HP offers additional advanced QM/MM functionalities with polarizable force fields. The continuous investigation efforts in our groups have the objective to bring sampling capabilities in a multiscale polarizable environment dedicated to electronic structure as sampling has been shown to be a key issue for predictive studies.^80^

## 7 Conclusion and perspectives

Our results demonstrate that molecular dynamics simulations with advanced point dipole polarizable force fields using distributed multipoles should no longer be qualified as slow anymore. The Tinker-HP software offers an efficient environment that enables one to perform large scale relatively long MD simulations on various complex systems encompassing several million atoms thanks to the new extension of 3D spatial decomposition to polarizable models coupled to advanced Krylov polarization solvers. It is able to ensure accuracy and speed as it exploits double precision, thanks to its new algorithmics able to circumvent the computational burden providing both additional speedups and mathematical robustness. For small systems, Tinker-HP is competitive with the present GPU implementation of Tinker (Tinker-OpenMM) whereas strong gains are observed for medium systems offering several thousand-fold acceleration compared to single core computations. For large systems, Tinker-HP remains the only operational Tinker code as it is able to efficiently distribute memory among nodes. We believe that this new tool will be of interest for the community of modelers, who will be able to perform meaningful simulations to test the applicability and discuss advantages of polarizable potentials. Of course, such developments will first find an echo in the field of chemistry where extreme accuracy matters, for example using embeddings of QM methods by PFFS that are beneficial to compute properties and where double precision is mandatory. For biophysics, where extreme sampling is required, the full application of PFFs remains a daunting task as present AMOEBA simulations, despite the discussed acceleration on large systems, still require weeks of computation. However, a few microseconds simulations are now technically possible and some applications such as free energy computations are completely accessible. In some way, PFFs are now able to produce simulations that classical force fields were able to generate a few years ago on similar platforms. The one-order of magnitude difference in speed of PFFs compared to classical FFs (when one considers the same computational platform, *i.e.* CPU or GPU), will remain due to the lower functional form complexity of the latter. However, the acceleration gains observed in optimal timings for codes like AMBER, NAMD, GROMACS or equivalent, are all obtained using GPU accelerators and through many years of optimization. Still, an important point to evaluate the future of PFF simulations is the fact that we have been really conservative in our present discussed benchmarks and optimization is only starting. Issues of precision, cutoffs, convergence criteria and vectorization will be addressed and will generate strongly improved performances. Note that the Tinker-HP methodology is not limited to CPUs. Indeed, the Tinker-HP FORTRAN legacy code will benefit from GPU acceleration as FORTRAN portability strategies exist and are under investigation (Hybrid-Fortran^94^ and OpenACC^95^). For CPUs, we also expect strong performance gains on new generation “big core” Xeon (Skylake and successors) and “small core” Xeon-Phi (Knight Landings) processors thanks to vectorization efforts exploiting AVX512 instructions without sacrificing double precision. Finally, Tinker-HP will be synchronized with Tinker-OpenMM^34^ opening our developments to the OpenMM community. Various method developments, already present in the Tinker community, will be integrated in the next version of the code, keeping in mind the mandatory philosophy to include only well-understood and scalable techniques. The high-performance implementation of additional multipolar polarizable force fields will be performed including the SIBFA^26^ (in progress), MPID^96^ (multipole and induced dipoles, the mapping of the CHARMM Drude polarizable force field on induced dipoles) and AMOEBA 2 models. Efforts will also be devoted to the porting of the third generation GEM (Gaussian Electrostatic Model) polarizable force field that relies on frozen distributed densities.^30^,^97^,^98^ The present technology will be complemented by massively parallel Monte-Carlo approaches, Langevin, constant-pH and various types of accelerated molecular dynamics. Advanced sampling techniques such as OSRW^74^ and replica exchange will be added. Concerning multiscale QM/MM simulations, studies towards coupling with linear scaling QM approaches will be pursued to continue to speed up hybrid MD simulations.

## Conflicts of interest

There are no conflicts to declare.

## Supplementary Material

Supplementary information
