# Supplementary material for: Tinker-HP: a massively parallel molecular dynamics package for multiscale simulations of large complex systems with advanced point dipole polarizable force fields
Source: Chem Sci. 2017 Nov 27;9(4):956–72. doi: 10.1039/c7sc04531j (PMC5909332; doi:10.1039/c7sc04531j)

Electronic Supplementary Material (ESI) for Chemical Science.
This journal is © The Royal Society of Chemistry 2017

Tinker-HP: a Massively Parallel Molecular Dynamics Package for Multiscale Simulations of Large Complex Systems with Advanced point Dipole Polarizable Force Fields

Supplementary Informations

S1

Table 1: Comparison of hydration free energies calculated from Tinker-HP vs. previous works.

	Hydration free energy (kcal/mol)			
	Water	Benzene	Sodium	Potassium
TinkerHP, BAR	-5.72 (0.10)	-1.27 (0.11)	-89.66 (0.15)	-71.56 (0.12)
Tinker, BAR	-5.86 (0.19)	-1.23 (0.23)	-89.9 (0.1)	-72.6 (0.1)
Tinker, OSRW			-88.9	-71.7

i) A. Grossfield, P. Ren and J. W. Ponder, J. Am. Chem. Soc., **2003**, 125, 15671-15682

ii) J. R. Abella, S. Y. Cheng, Q. Wang, W. Yang, Pengyu Ren, J. Chem. Theory Comput**.**, **2014**, 10, 2792–2801

S2

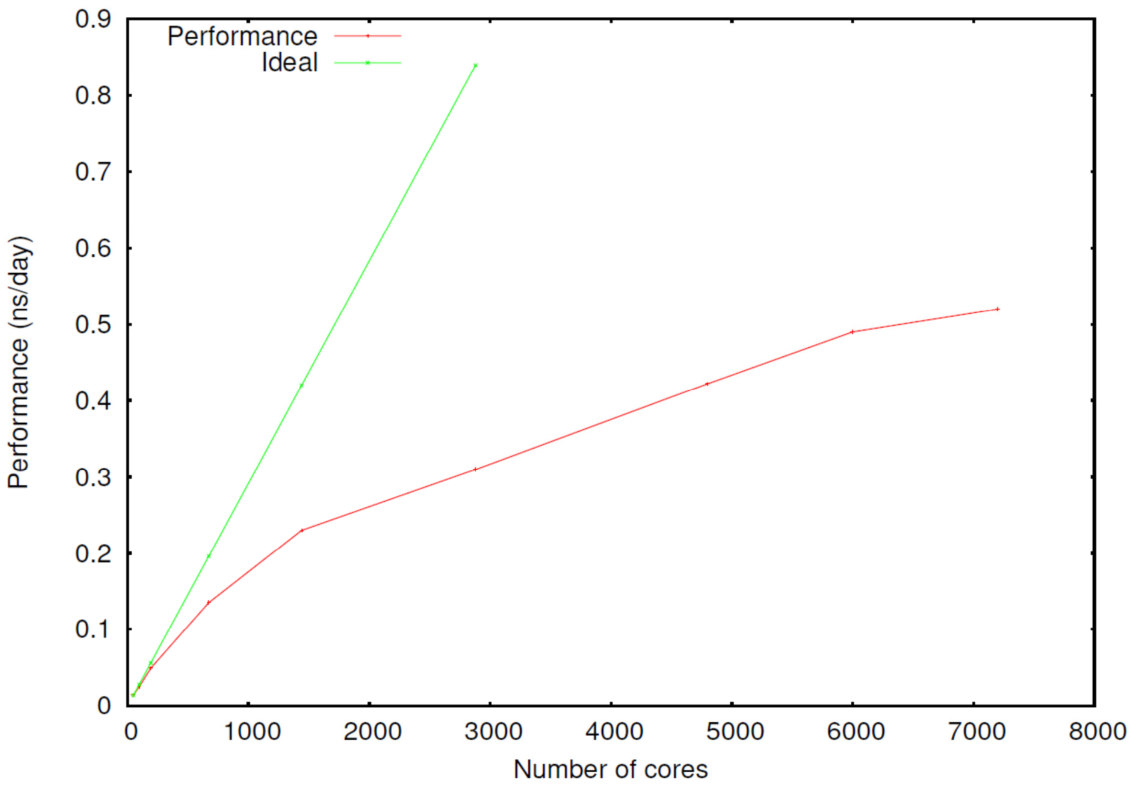

Figure S1: Performance gain for the Lake water box vs. ideal scaling

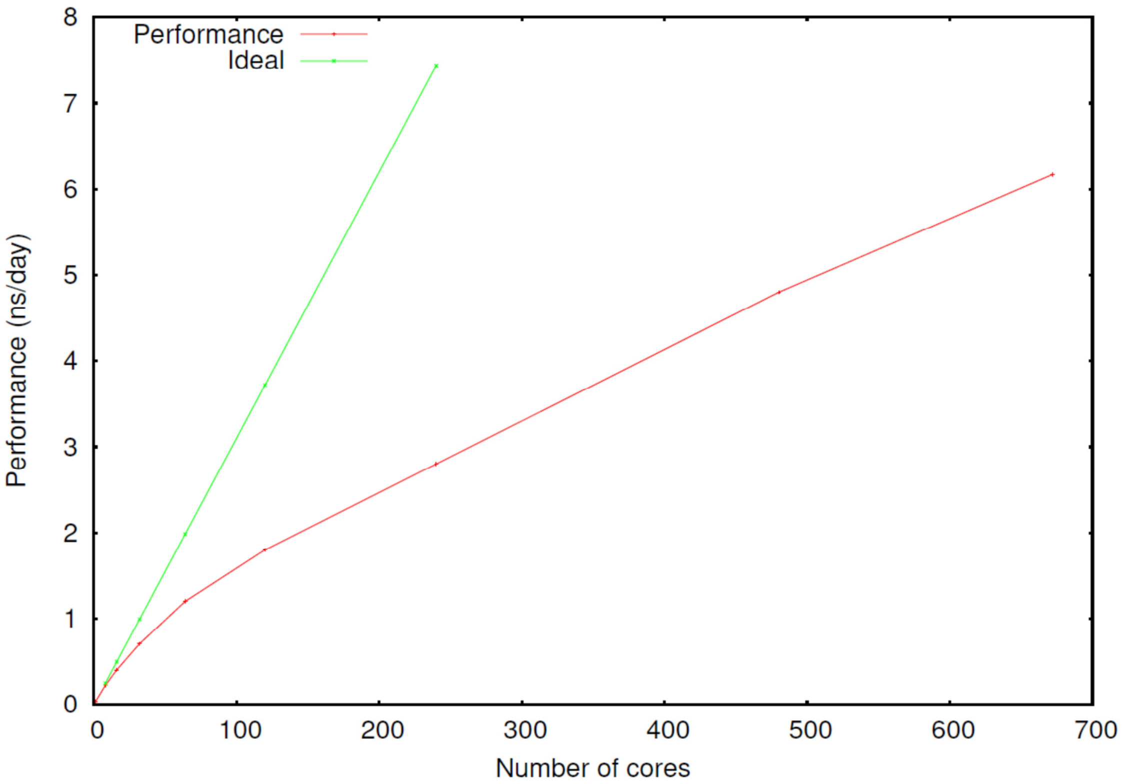

Figure S2: Performance gain for the DHFR protein vs. ideal scaling

S3

Tinker-HP scaling results for the ddCosmo approach with AMOEBA

The computations have been performed with really conservative setups:

Parameters for ddCOSMO :
lmax (maximum angular momentum of the spherical harmonics basis) : 8
ndgrid (Number of points for the Lebedev grid) : 194
Convergence criterion: 10^{-6}
eps (dielectric permitivity) : 78.3553
eta (switching parameter) : 0.2

See the following reference for detailed explanations:
F. Lipparini, L. Lagardère, C. Raynaud, B. Stamm, E. Cances, M. Schnieders, P. Y. Ren, B. Mennucci, Y. Maday, J.-P. Piquemal, J. Chem. Theory. Comput., **2015**, 11, 623-634

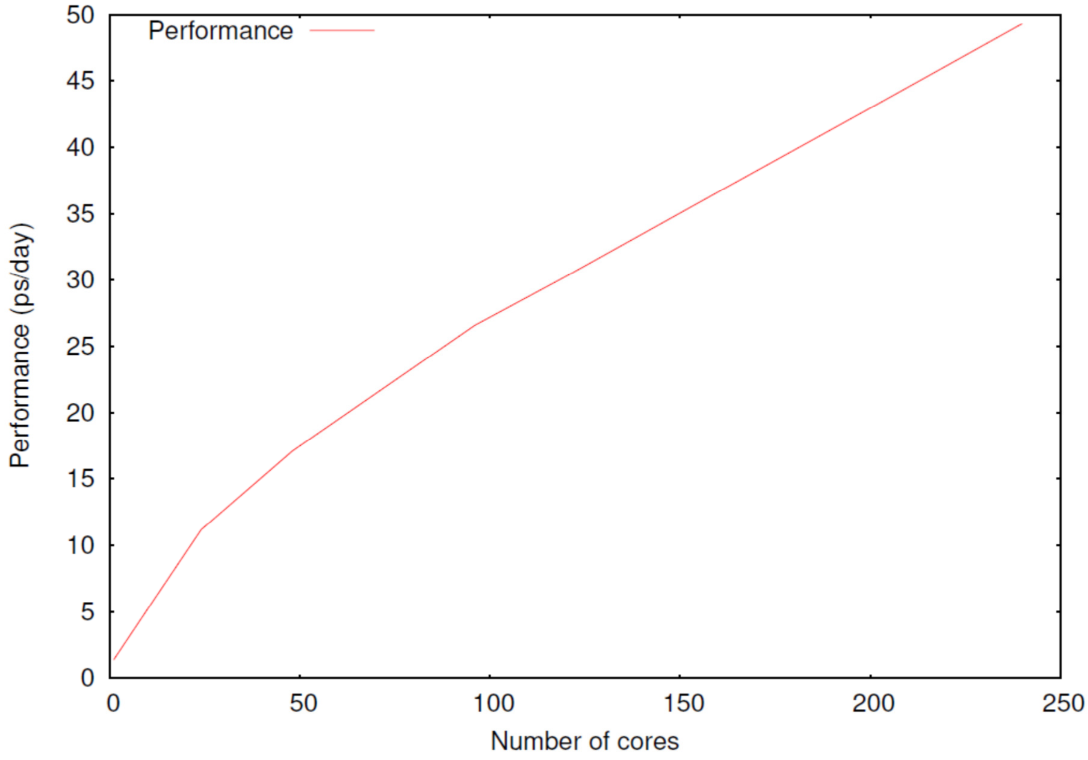

Figure S3: Performance gain for the ubiquitin protein (1232 atoms) with ddCOSMO

Figure S4: Performance gain for the DHFR protein (2489 atoms) with ddCOSMO

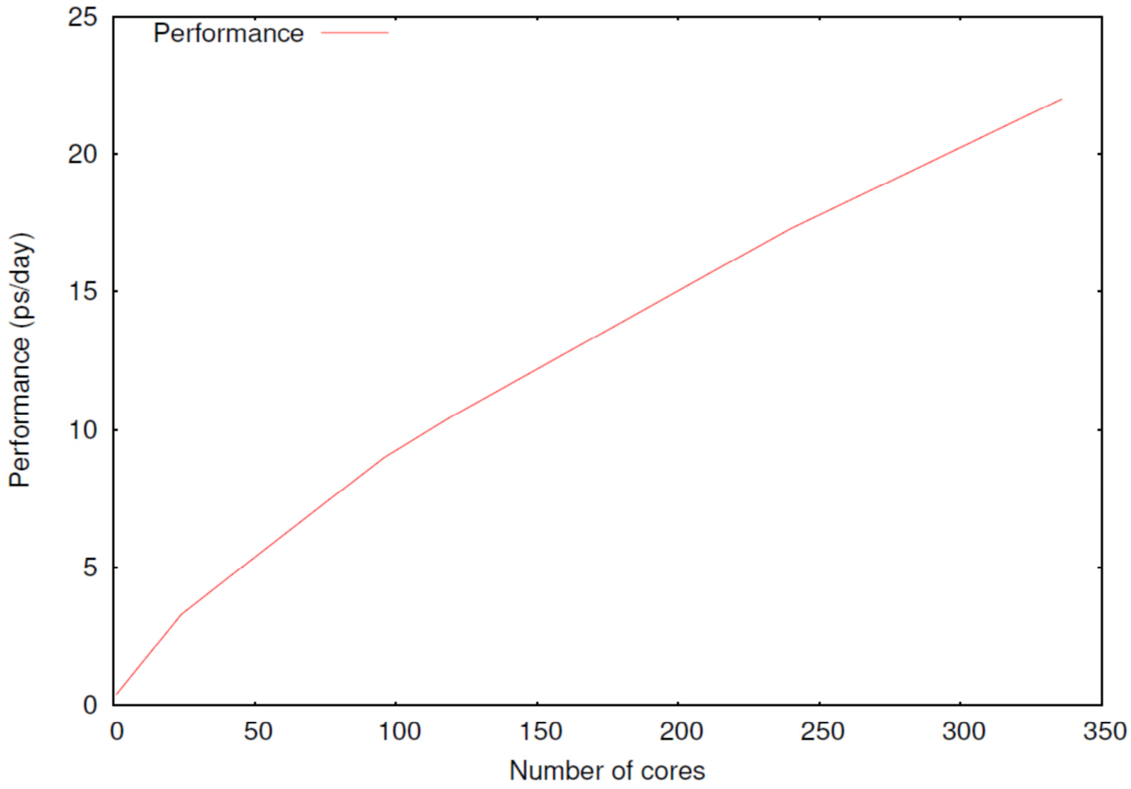

Supplement: Supplementary file 1 [file SC-009-C7SC04531J-s001.pdf]
